# A Comparison of Personalized and Generalized Approaches to Emotion Recognition Using Consumer Wearable Devices: Machine Learning Study

**DOI:** 10.2196/52171

**Published:** 2024-05-10

**Authors:** Joe Li, Peter Washington

**Affiliations:** 1 Information and Computer Sciences University of Hawai`i at Mānoa Honolulu, HI United States

**Keywords:** affect detection, affective computing, deep learning, digital health, emotion recognition, machine learning, mental health, personalization, stress detection, wearable technology

## Abstract

**Background:**

There are a wide range of potential adverse health effects, ranging from headaches to cardiovascular disease, associated with long-term negative emotions and chronic stress. Because many indicators of stress are imperceptible to observers, the early detection of stress remains a pressing medical need, as it can enable early intervention. Physiological signals offer a noninvasive method for monitoring affective states and are recorded by a growing number of commercially available wearables.

**Objective:**

We aim to study the differences between personalized and generalized machine learning models for 3-class emotion classification (neutral, stress, and amusement) using wearable biosignal data.

**Methods:**

We developed a neural network for the 3-class emotion classification problem using data from the Wearable Stress and Affect Detection (WESAD) data set, a multimodal data set with physiological signals from 15 participants. We compared the results between a participant-exclusive generalized, a participant-inclusive generalized, and a personalized deep learning model.

**Results:**

For the 3-class classification problem, our personalized model achieved an average accuracy of 95.06% and an *F*_1_-score of 91.71%; our participant-inclusive generalized model achieved an average accuracy of 66.95% and an *F*_1_-score of 42.50%; and our participant-exclusive generalized model achieved an average accuracy of 67.65% and an *F*_1_-score of 43.05%.

**Conclusions:**

Our results emphasize the need for increased research in personalized emotion recognition models given that they outperform generalized models in certain contexts. We also demonstrate that personalized machine learning models for emotion classification are viable and can achieve high performance.

## Introduction

Stress and negative affect can have long-term consequences for physical and mental health, such as chronic illness, higher mortality rates, and major depression [1-3]. Therefore, the early detection and corresponding intervention of stress and negative emotions greatly reduces the risk of detrimental health conditions appearing later in life [4]. Since negative stress and affect can be difficult for humans to observe [5-7], automated emotion recognition models can play an important role in health care. Affective computing can also facilitate digital therapy and advance the development of assistive technologies for autism [8-13].

Physiological signals, including electrocardiography (ECG), electrodermal activity (EDA), and photoplethysmography (PPG), have been shown to be robust indicators of emotions [14-16]. The noninvasive nature of physiological signal measurement makes it a practical and convenient method for emotion recognition. Wearable devices such as smartwatches have become increasingly popular, and products such as Fitbit have already integrated the sensing of heart rate, ECG, and EDA data into their smartwatches. The accessibility of wearable devices indicates that an emotion recognition model using biosignals can have practical applications in health care.

The vast majority of research in recognizing emotions from biosignals involves machine learning models that are generalizable, which means that the models were trained on one group of subjects and tested on a separate group of subjects [17-28]. Prior studies emphasize the need for personalized or subject-dependent models [18,29,30], and some investigations, albeit few, analyze personalized models [31,32]. Both generalized and personalized models have potential benefits; for example, generalized models can train on more data than personalized models, and personalized models do not need to address the problem of inter-subject data variance [33]. However, it is still unclear how personalized models compare against generalized models in many contexts.

We present 1 personalized and 2 generalized machine learning approaches for the 3-class emotion classification problem (neutral, stress, and amusement) on the Wearable Stress and Affect Detection (WESAD) data set, a publicly available data set that includes both stress and emotion data [18]. The two generalized models are trained using participant-inclusive and participant-exclusive procedures. We compare the performance of these 3 models, finding that the personalized machine learning approach consistently outperforms the generalized approach on the WESAD data set.

## Methods

### Overview

**Figure 1:**
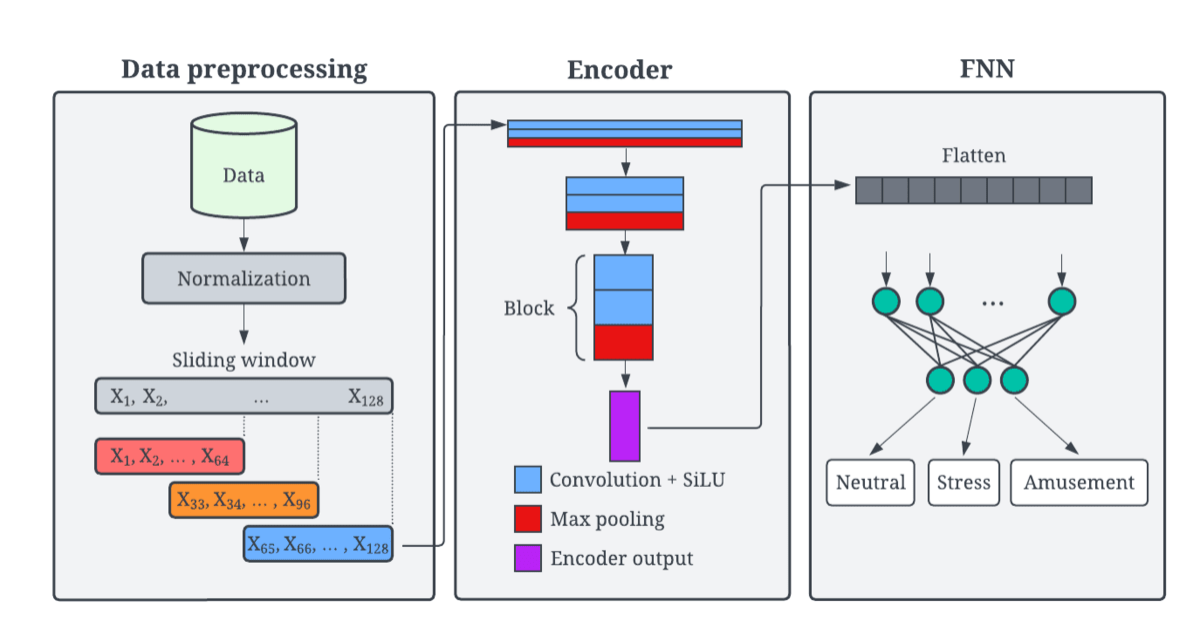
Overview of our model architecture for the 3-class emotion classification task. FNN: feedforward neural network; SiLU: sigmoid linear unit.

To classify physiological data into the neutral, stress, and amusement classes, we developed a machine learning framework and evaluated the framework using data from the WESAD data set. Our machine learning framework consists of data preprocessing, a convolutional encoder for feature extraction, and a feedforward neural network for supervised prediction (Figure 1). Using this model architecture, we compared generalized and personalized approaches to the 3-class emotion classification task (neutral, stress, and amusement).

### Data Set

We selected WESAD, a publicly available data set that combines both stress and emotion annotations. WESAD consists of multimodal physiological data in the form of continuous time-series data for 15 participants and corresponding annotations of 4 affective states: neutral, stress, amusement, and meditation. However, we only considered the neutral, stress, and amusement classes since the objective of WESAD is to provide data for the 3-class classification problem, and the benchmark model in WESAD ignores the meditation state as well. Our model incorporated data from 8 modalities recorded in WESAD: ECG, EDA, electromyogram (EMG), respiration, temperature, and acceleration (x, y, and z axes). In the data set, measurements for each of the 8 modalities were sampled by a RespiBAN sensor at 700 Hz to enforce uniformity, and data were collected for approximately 36 minutes per participant.

### Preprocessing and Partitioning

Each data modality was normalized with a mean of 0 and an SD of 1. We used a sliding window algorithm to partition each modality into intervals consisting of 64 data points, with a 50% overlap between consecutive intervals. We ensured that all 64 data points within an interval shared a common annotation, which allowed us to assign a single affective state to each interval. The process of normalization, followed by a sliding window partition, is illustrated in Figure 1. These intervals were partitioned into training, validation, and testing sets.

**Figure 2:**
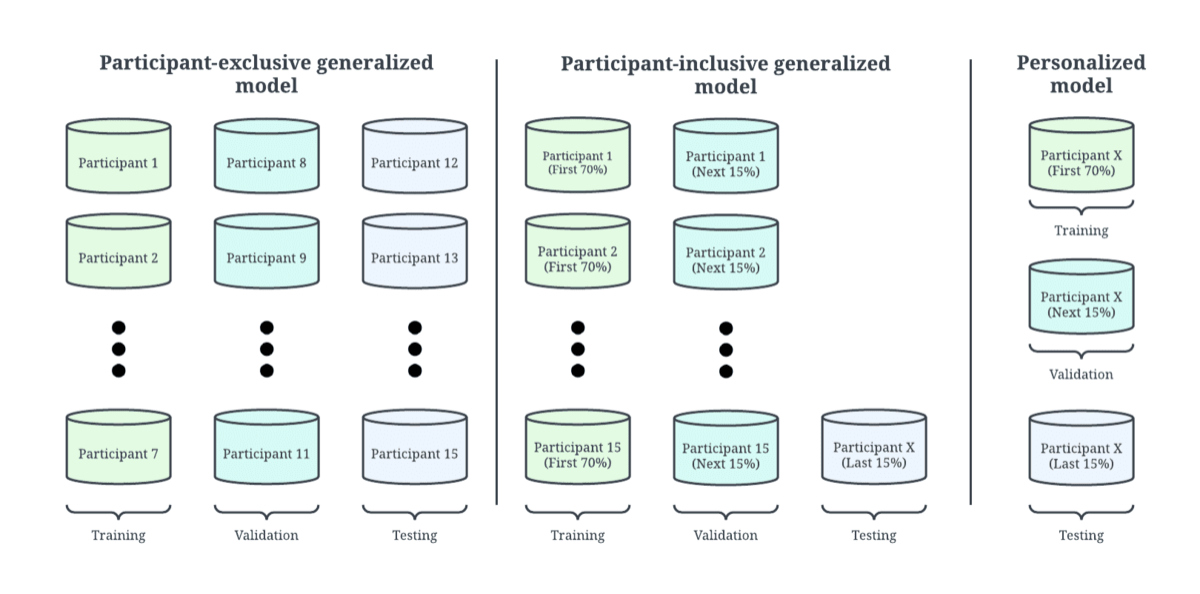
A comparison of different generalized and personalized approaches to the 3-class emotion classification task. The participant-exclusive generalized model mimics generalized approaches used in other papers. The participant-exclusive generalized model shown in the figure differs from what we use in this paper.

For the personalized model, we partitioned the training, validation, and testing sets as follows: each participant in the data set had their own model that was trained, validated, and tested independently of other participants. For each affective state (neutral, stress, and amusement), we allocated the initial 70% of intervals with that affective state for training, the next 15% for validation, and the final 15% for testing. This guaranteed that the relative frequencies of each affective state were consistent across all 3 sets. Simply using the first 70% of all intervals for the training data would skew the distribution of affective states, given the nature of the WESAD data set. Furthermore, our partitioning of intervals according to sequential time order rather than random selection helped prevent overfitting by guaranteeing that 2 adjacent intervals with similar features would be in the same set. The partitioning of training, validation, and testing sets for the personalized model is shown in Figure 2.

Standard generalized models partition the training, validation, and testing sets by participant [18]. We denote these standard models as participant-exclusive generalized models, as shown in Figure 2. Through this partitioning method, it is impossible to compare the performances of generalized and personalized models since they are solving two separate tasks. Therefore, we present a modified participant-exclusive generalized model that solves the same task as the personalized model. The testing set for our participant-exclusive generalized model consisted of the last 15% of intervals for each affective state for 1 participant. The training set consisted of the first 70% of intervals for each affective state for all participants except the 1 participant in the testing set, and the validation set consisted of the next 15% of intervals for all participants except the 1 participant in the testing set. The training and testing sets for this approach contained data from mutually exclusive sets of participants; this is where the name of the model, participant-exclusive, is derived from. Since the testing sets for the participant-exclusive generalized and personalized models are equivalent, it is possible to compare generalized and personalized approaches. This participant-exclusive generalized model served as our first generalized model baseline.

A second generalized model baseline was created, called the participant-inclusive generalized model. Like the testing sets for the participant-exclusive generalized and personalized models, the testing set for this model contained the last 15% of intervals for each affective state for a single participant. The training set consisted of the first 70% of intervals for each affective state for all participants, and the validation set consisted of the next 15%. The set of participants in the training and testing sets overlapped by 1 participant—the subject in the testing set—which is why this model is called the participant-inclusive generalized model. This is illustrated in Figure 2.

### Model Architecture

The model architecture consisted of an encoder network followed by a feedforward head, which is shown in Figure 1. A total of 8 channels, representing the 8 modalities we used from WESAD, served as input into an encoder network, which was modeled after the encoder section of U-Net [34]. The encoder network had 3 blocks, with each block consisting of two 1D convolutional layers (kernel size of 3) followed by 1D max pooling (kernel size of 2). The output of each convolution operation was passed through a sigmoid linear unit (SiLU) activation function. Between each block, we doubled the number of channels and added a dropout layer (15%) to reduce overfitting. The output of the encoder was flattened and passed through 2 fully connected layers with SiLU activation to produce a 3-class probability distribution. Table 1 shows the hyperparameters that determine the model structure. These were consistent between the participant-exclusive generalized, participant-inclusive generalized, and personalized models.

**Table 1:** Hyperparameters relating to model structure.

Hyperparameter	Value
Encoder depth (number of blocks), n	3
Dropout rate, %	15
Number of fully connected layers, n	2
Convolutional kernel size, n	3
Max pooling kernel size, n	2
Activation function	SiLU^a^

^a^SiLU: sigmoid linear unit.

### Model Training

We trained the 2 generalized baseline models and the personalized model under the same hyperparameters to guarantee a fair comparison. Both models were trained with cross-entropy loss using AdamW optimization. All models were written using PyTorch [35]. Within 1000 epochs, models with the lowest validation loss were saved for testing. A Nvidia GeForce RTX 4090 GPU was used for training. A separate personalized model was trained for each of the 15 participants. The participant-exclusive generalized model was trained 15 times, and the participant-inclusive generalized model was trained once. For model comparison, all models were tested on each of the 15 participants.

### Ethical Considerations

This study did not require institutional review board (IRB) review because we exclusively used a commonly analyzed publicly available data set. We did not work with any human subjects.

## Results

For the 3-class emotion classification task (neutral, stress, and amusement), Tables 2 and 3 illustrate the accuracy and *F*_1_-score of the personalized and generalized models when tested on each of the 15 participants. We include *F*_1_-score, a balanced evaluation metric consisting of the harmonic mean of precision and recall, to accommodate for the imbalanced class distribution in WESAD [18]. In order to guarantee a fair comparison between the models, they had the same random seeds for model initialization, and their architecture and hyperparameters were the same. The accuracy and *F*_1_-score for the personalized model exceeded those of the participant-inclusive generalized model for all participants except participant 1, and the personalized model outperformed the participant-exclusive generalized model in terms of accuracy and *F*_1_-score for all participants. The personalized models for participants 1 and 2 also indicate subpar performance compared to other participants, which we address in the Discussion section.

Table 4 shows the average and SD of the accuracies and *F*_1_-scores across all participants for the 3 models. We achieved an average accuracy of 95.06%, 66.95%, and 67.65% for the personalized, participant-inclusive generalized, and participant-exclusive generalized models, respectively. We also achieved an average *F*_1_-score of 91.72%, 42.50%, and 43.05% for the personalized, participant-inclusive generalized, and participant-exclusive generalized models, respectively. Observing the error margins in Table 4, the differences in accuracy and *F*_1_-score between the personalized model and both generalized models are statistically significant. As shown in Table 5, we evaluated the *P* values between each model type for accuracy and *F*_1_-score through pairwise 2-tailed *t* tests to determine statistical significance.

**Table 2:** A comparison of model accuracy between the personalized and generalized models.

Participant	Model accuracy, %		
	Personalized model	Participant-inclusive generalized model	Participant-exclusive generalized model
1	68.36	82.69	53.94
2	82.32	67.12	81.91
3	99.99	82.81	82.81
4	99.90	82.86	82.31
5	98.02	82.94	74.67
6	99.57	54.57	54.03
7	100.00	82.05	83.23
8	100.00	53.72	53.70
9	100.00	51.86	51.83
10	93.69	82.05	79.85
11	100.00	60.86	62.11
12	98.34	53.53	53.60
13	99.81	53.26	65.35
14	100.00	53.47	53.54
15	85.83	60.43	81.91

**Table 3:** A comparison of F_1_-score between the personalized and generalized models.

Participant	*F*_1_-score, %		
	Personalized model	Participant-inclusive generalized model	Participant-exclusive generalized model
1	58.14	61.91	23.36
2	58.88	44.55	58.53
3	99.98	62.05	62.05
4	99.87	61.95	61.50
5	96.87	61.99	54.74
6	99.35	24.94	23.59
7	100.00	61.16	62.09
8	100.00	23.38	23.29
9	100.00	22.85	22.89
10	94.29	61.04	59.23
11	100.00	38.27	40.15
12	97.40	26.79	26.90
13	99.75	24.47	44.63
14	100.00	23.93	24.09
15	71.28	38.26	58.71

**Table 4:** Average accuracy and F_1_-score of models across all participants.

Model type	Accuracy, mean (SD [%])	*F*_1_-score, mean (SD [%])
Personalized	95.06 (9.24)	91.72 (15.33)
Participant-inclusive generalized	66.95 (13.76)	42.50 (17.37)
Participant-exclusive generalized	67.65 (13.48)	43.05 (17.20)

**Table 5:** *P* values of accuracy and F_1_-score comparisons between model types.

Model comparison	*P* value for accuracy	*P* value for *F*_1_-score
Personalized versus participant-inclusive generalized	*P<*.001	*P<*.001
Personalized versus participant-exclusive generalized	*P<*.001	*P<*.001
Participant-inclusive generalized versus participant-exclusive generalized	.81	.88

## Discussion

### Principal Findings

We demonstrated that a personalized deep learning model outperforms a generalized model in both the accuracy and *F*_1_-score metrics for the 3-class emotion classification task. By establishing two generalized model baselines through the participant-inclusive and participant-exclusive models, we created an alternative approach to the standard generalization technique of separating the training and testing sets by participant, and as a result, we were able to compare personalized and generalized approaches. Our personalized model achieved an accuracy of 95.06% and an *F*_1_-score of 91.72%, while our participant-inclusive generalized model achieved an accuracy of 66.95% and an *F*_1_-score of 42.50% and our participant-exclusive generalized model achieved an accuracy of 67.65% and an *F*_1_-score of 43.05%.

Our work indicates that personalized models for emotion recognition should be further explored in the realm of health care. Machine learning methods for emotion classification are clearly viable and can achieve high accuracy, as shown by our personalized model. Furthermore, given that numerous wearable technologies collect physiological signals, data acquisition is both straightforward and noninvasive. Combined with the popularity of consumer wearable technology, it is feasible to scale emotion recognition systems. This can ultimately play a major role in the early detection of stress and negative emotions, thus serving as a preventative measure for serious health problems.

### Comparison With Previous Work

#### Generalized Models

The vast majority of prior studies using WESAD developed generalized approaches to the emotion classification task. Schmidt et al [18], the pioneers of WESAD, created several feature extraction models and achieved accuracies up to 80% for the 3-class classification task. Huynh et al [22] developed a deep neural network, trained on WESAD wrist signals, to outperform past approaches by 8.22%. Albaladejo-González et al [36] achieved an *F*_1_-score of 88.89% using an unsupervised local outlier factor model and 99.03% using a supervised multilayer perceptron. Additionally, they analyzed the transfer learning capabilities of different models between the WESAD and SWELL-KW (SWELL knowledge work) [37] data sets. Ghosh et al [38] achieved 94.8% accuracy using WESAD chest data by encoding time-series data into Gramian Angular Field images and employing deep learning techniques. Bajpai et al [39] investigated the k-nearest neighbor algorithm to explore the tradeoff between performance and the total number of nearest neighbors using WESAD. Through federated learning, Almadhor et al [40] achieved 86.82% accuracy on data in WESAD using a deep neural network. Behinaein et al [41] developed a novel transformer approach and achieved state-of-the-art performance using only one modality from WESAD.

#### Personalized Models

Sah and Ghasemzadeh [30] developed a generalized approach using a convolutional neural network using 1 modality from WESAD. For the 3-class classification problem, they achieved an average accuracy of 92.85%. They used the leave-one-subject-out (LOSO) analysis to highlight the need for personalization. Indikawati and Winiarti [31] directly developed a personalized approach for the 4-class classification problem in WESAD (neutral, stress, amusement, and meditation). Using different feature extraction machine learning models, they achieved accuracies ranging from 88%-99% for the 15 participants. Liu et al [32] developed a federated learning approach using data from WESAD with the goal of preserving user privacy. In doing so, they developed a personalized model as a baseline, which achieved an average accuracy of 90.2%. Nkurikiyeyezu et al [42] determined that personalized models (95.2% accuracy) outperform generalized models (42.5% accuracy) for the stress versus no-stress task. By running additional experiments to further understand how personalized models compare to generalized models for the 3-class emotion classification task and by developing participant-inclusive and participant-exclusive versions of the generalized models, our work concretely demonstrates how personalization outperforms generalization and thus supports the conclusions of Nkurikiyeyezu et al [42].

### Limitations and Future Work

**Figure 3:**
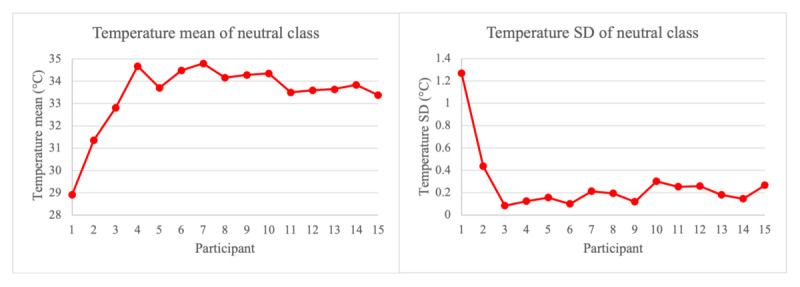
Deviations of mean and SD for participants 1 and 2 for neutral class modalities.

**Figure 4:**
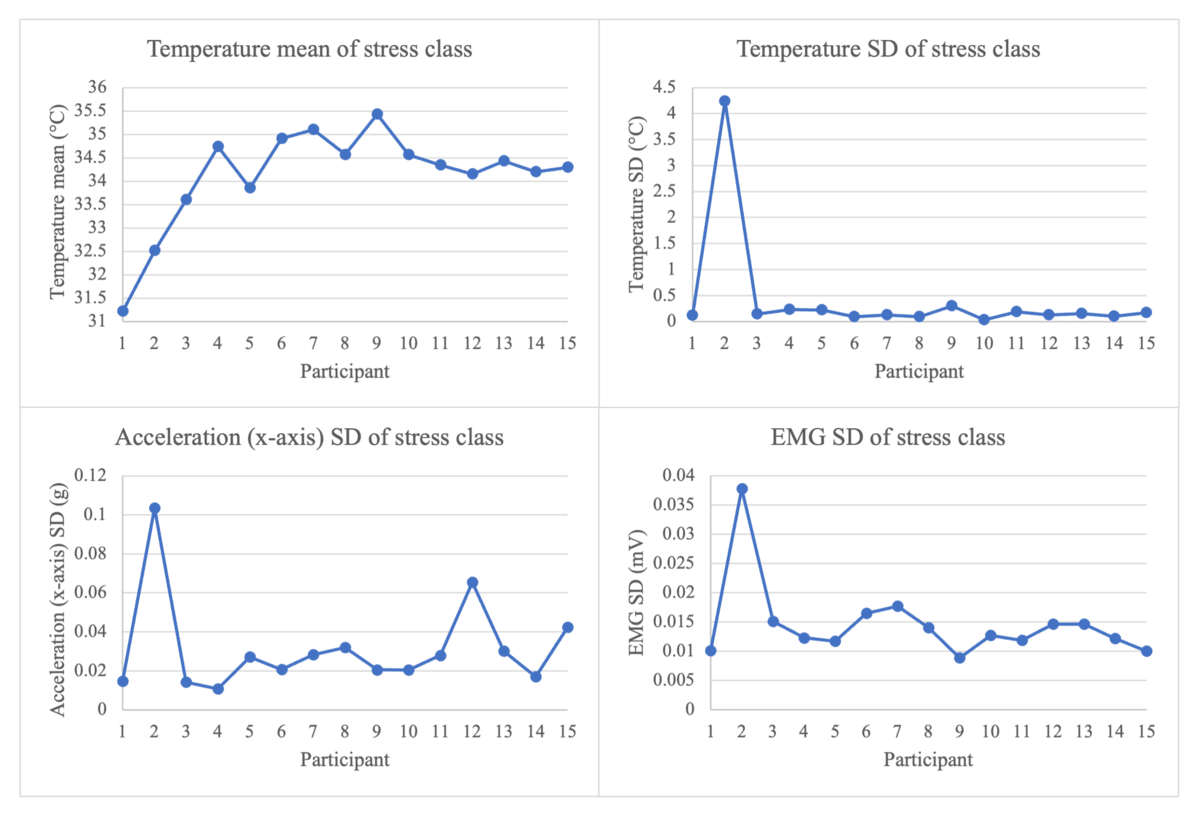
Deviations of mean and SD for subjects 1 and 2 for stress class modalities. EMG: electromyogram.

**Figure 5:**
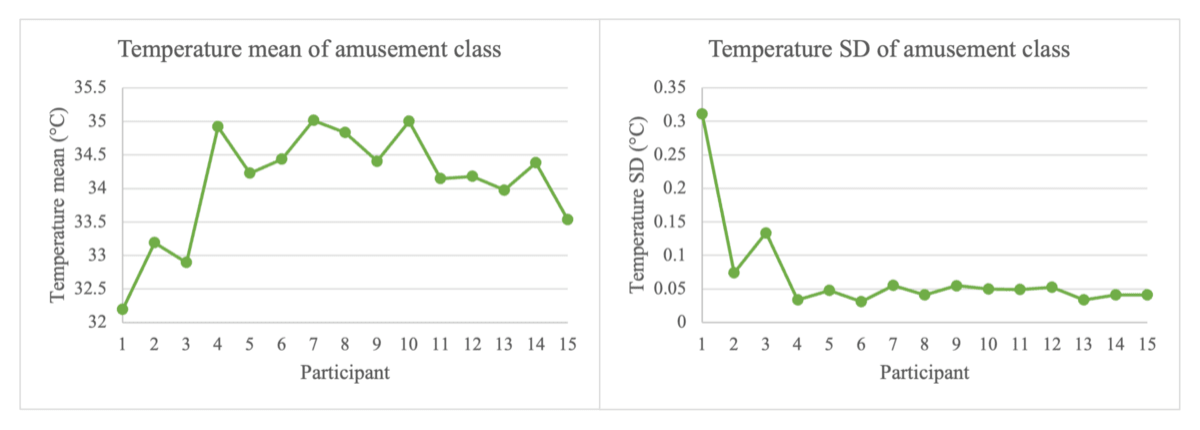
Deviations of mean and SD for subjects 1 and 2 for amusement class modalities.

As shown in Tables 2 and 3, the performance of our personalized model deteriorates for participants 1 and 2. To analyze the lack of performance improvement of the personalized model for these 2 participants, we visualized the means and SDs of the different modalities for each emotion class. In Figures 3-5, we illustrate notable deviations in modality means and SDs for participants 1 and 2 compared to other participants. While the analysis of these modalities reveals important information about the nature of the WESAD data set, it still remains difficult to pinpoint the exact data set features that caused the performance decline in the personalized model for these 2 participants. This is another limitation: since we do not use a feature extraction model, we cannot assign a feature importance (eg, Gini importance) to individual features like Schmidt et al [18] do. We also analyzed the emotion class balances for each participant, which are included in Table 6, to see if anomalies existed in the class distributions for certain participants. However, based on the ranges of the class distributions, class balance likely had minimal effect on the performance decline.

**Table 6:** Ranges of emotion class distributions per participant.

Emotion class	Range, %
Neutral	51.8-54.0
Stress	29.0-31.8
Amusement	16.3-17.4

Our participant-inclusive and participant-exclusive generalized models do not outperform previously published generalized models on the WESAD data set (eg, Schmidt et al [18] achieved up to 80% accuracy while we achieved 66.95% accuracy with our participant-inclusive model). This discrepancy can be attributed to a deliberate choice in our methodology: instead of maximizing our generalized models’ performance with hyperparameter tuning, we simply opted for a consistent set of hyperparameters across the personalized and generalized models because our primary objective was to evaluate their relative performance. While hyperparameter tuning might yield higher results in practice, differing hyperparameters between our models would introduce additional variables that make it difficult to determine the role that personalization and generalization play in model performance.

Given the variations between participants, one approach to improving generalized model performance is adding embedding representations for each participant or participant-specific demographic data as additional features as a method of distinguishing individual participants in generalized models. However, to prevent overfitting to participant-specific features like demographic data, data sets with significantly more participants would need to be created, given the small sample size of the WESAD data set.

One limitation that personalized models may encounter during training is the cold start problem, given that personalized models receive less data than generalized models. Moreover, despite the accuracy improvement in personalized models, developing a model for each participant may be costly and unscalable: data must be labeled specifically per participant, and enough data must be provided to the model to overcome the cold start problem (notably, however, even though the cold start problem should theoretically put our personalized model at a disadvantage, the WESAD data set provided enough data for our personalized model to outperform our generalized model). Both of these limitations can be addressed by a self-supervised learning approach to emotion recognition.

A self-supervised learning approach follows a framework used by natural language processing models such as the Bidirectional Encoder Representations from Transformers (BERT) model [43]. A model first pretrains on a large set of unlabeled data across numerous participants. Then, the pretrained model is fine-tuned to a small amount of labeled, participant-specific data. The pretraining phase eliminates the burden of manual labeling because all data are unlabeled, as well as the cold start problem because large amounts of data can be provided. The fine-tuning phase requires only a small amount of user-specific labeled data to perform accurately, and studies have already begun exploring the tradeoffs between the number of labels and model accuracy in WESAD using self-supervised or semisupervised approaches [44,45].

Finally, to expand beyond the WESAD data set, it is valuable to reproduce results on additional physiological signal data sets for emotion analysis, such as the Database for Emotion Analysis using Physiological Signals (DEAP) [46] and Cognitive Load, Affect, and Stress (CLAS) [47]. Data from WESAD were collected under controlled laboratory environments, which may not generalize to the real world. Therefore, analyzing emotions in a real-world context through data sets such as K-EmoCon [48], which contain physiological data collected in naturalistic conversations, may be useful. Emotions in the K-EmoCon data set were categorized into 18 different classes, so exploring this data set could also help us better assess the benefits of personalization for a broader range of emotions. A major goal of this approach is to provide support for personalized digital interventions for neuropsychiatry, which could benefit a variety of applications, such as video-based digital therapeutics for children with autism to predict the child’s affective state as part of the therapeutic process [49-52].

## Abbreviations

BERT: Bidirectional Encoder Representations from Transformers
CLAS: Cognitive Load, Affect, and Stress
DEAP: Database for Emotion Analysis using Physiological Signals
ECG: electrocardiography
EDA: electrodermal activity
EMG: electromyogram
LOSO: leave-one-subject-out
PPG: photoplethysmography
SiLU: sigmoid linear unit
SWELL: Smart Reasoning for Well-being at Home and at Work
SWELL-KW: SWELL knowledge work
WESAD: Wearable Stress and Affect Dataset
